# Profiling metabolites and lipoproteins in COMETA, an Italian cohort of COVID-19 patients

**DOI:** 10.1371/journal.ppat.1010443

**Published:** 2022-04-21

**Authors:** Veronica Ghini, Gaia Meoni, Lorenzo Pelagatti, Tommaso Celli, Francesca Veneziani, Fabrizia Petrucci, Vieri Vannucchi, Laura Bertini, Claudio Luchinat, Giancarlo Landini, Paola Turano

**Affiliations:** 1 Department of Chemistry, University of Florence, Sesto Fiorentino, Italy; 2 Magnetic Resonance Center (CERM), University of Florence, Sesto Fiorentino, Italy; 3 Consorzio Interuniversitario Risonanze Magnetiche di Metallo Proteine (CIRMMP), Sesto Fiorentino, Italy; 4 Internal Medicine, Santa Maria Nuova Hospital, Florence, Italy; 5 Laboratory of Clinical Pathology, Santa Maria Nuova Hospital, Florence, Italy; 6 Laboratory of Clinical Pathology, San Giovanni di Dio Hospital, Florence, Italy; University of Maryland, UNITED STATES

## Abstract

Metabolomics and lipidomics have been used in several studies to define the biochemical alterations induced by COVID-19 in comparison with healthy controls. Those studies highlighted the presence of a strong signature, attributable to both metabolites and lipoproteins/lipids. Here, ^1^H NMR spectra were acquired on EDTA-plasma from three groups of subjects: i) hospitalized COVID-19 positive patients (≤21 days from the first positive nasopharyngeal swab); ii) hospitalized COVID-19 positive patients (>21 days from the first positive nasopharyngeal swab); iii) subjects after 2–6 months from SARS-CoV-2 eradication. A Random Forest model built using the EDTA-plasma spectra of COVID-19 patients ≤21 days and Post COVID-19 subjects, provided a high discrimination accuracy (93.6%), indicating both the presence of a strong fingerprint of the acute infection and the substantial metabolic healing of Post COVID-19 subjects. The differences originate from significant alterations in the concentrations of 16 metabolites and 74 lipoprotein components. The model was then used to predict the spectra of COVID-19>21 days subjects. In this group, the metabolite levels are closer to those of the Post COVID-19 subjects than to those of the COVID-19≤21 days; the opposite occurs for the lipoproteins. Within the acute phase patients, characteristic trends in metabolite levels are observed as a function of the disease severity. The metabolites found altered in COVID-19≤21 days patients with respect to Post COVID-19 individuals overlap with acute infection biomarkers identified previously in comparison with healthy subjects. Along the trajectory towards healing, the metabolome reverts back to the “healthy” state faster than the lipoproteome.

## Author summary

^1^H NMR spectra of EDTA-plasma from 246 COVID-19-positive subjects in the acute phase of infection were compared to those of 95 COVID-19-recovered subjects. The two cohorts are largely different (discrimination accuracy > 93%) due to a pool of 16 metabolites and 74 lipoprotein parameters significantly up- or down-regulated in the patients and within the healthy range in the recovered subjects. In 28 post-acute COVID-19-positive patients, the metabolites levels are reverted back to normality whereas the lipoprotein parameters are still altered. Therefore, the metabolite biomarkers might be used as the timeliest sign of the individual response to treatment or spontaneous healing.

## Introduction

Coronavirus disease 2019 (COVID-19) is a viral pandemic caused by the severe acute respiratory syndrome coronavirus 2 (SARS-CoV-2). SARS-CoV-2 is single strand RNA virus, and its human-to human transmission occurs mainly via respiratory aerosols and droplets. The incubation period ranges from 1 to 14 days, in most cases 3–5 days [1,2]. The infection is characterized by a wide range of clinical manifestations; while many patients are asymptomatic or paucisymptomatic with flu-like symptoms, other rapidly develop interstitial pneumonia, acute respiratory distress syndrome (ARDS) and respiratory failure, requiring hospitalization and even ventilation [1,2], thus exerting enormous pressure on the national health systems. Although most COVID-19 patients primarily develop respiratory symptoms, a wide range of other symptoms and manifestations associated with COVID-19 have been observed, such as sepsis, thromboembolism and multi-organ disfunctions [3,4]. Additionally, a significant subset of patients, including mild or asymptomatic cases, are afterwards developing persistent symptoms indicated as Long COVID [5]. Omics sciences can contribute to monitoring this complex scenario and understanding its biological determinants.

Several metabolomic studies have indicated that COVID-19 is associated with abnormalities in the concentration of metabolites and lipoproteins in plasma or serum [6–20]. These findings suggest the reprogramming of some metabolic pathways alongside the reprogramming of the immune system. The COVID-19 signature at the level of the metabolome and lipoproteome is particularly strong and highly reproducible, even in studies conducted on cohorts of patients from different countries.

Among the possible analytical platforms, NMR-based metabolomics has the advantage of being highly reproducible, fast, and requiring minimal sample handling [21–24]. It is therefore compatible with medium/high-throughput applications. ^1^H NMR operates in untargeted mode and permits the simultaneous observation and quantification of all those metabolites that are present in concentration down to 1 μM, which in plasma and serum correspond to about 30 small organic molecules. With the same approach one can simultaneously quantify the lipoprotein class-specific concentrations of cholesterol, phospholipids and triglycerides, thus providing an exhaustive description of the lipoproteome in addition to the NMR-detectable fraction of the metabolome [25]. **S1 and S2 Figs** summarize the main results of NMR metabolomic studies on the serum or plasma of COVID-19 patients in comparison with control populations [6–14].

Here, we profiled via ^1^H NMR the metabolome and the lipoprotein fractions in the plasma of patients who sustain an acute SARS-CoV-2 infection with various disease severity and compared these profiles with those of the plasma of subjects in follow-up after 2–6 months from the first negative swab. The whole NMR spectrum, which embeds the metabolome and lipoproteome information, has a high discriminatory power between the two groups. The discrimination is essentially determined by alterations in the levels of those molecular components identified as biomarkers of the disease [6–14]. Indeed, their concentrations in the follow-up group are essentially within the normality range. Interestingly, the plasma of COVID-19 patients collected at > 21 days from the first positive swab has a metabolomics fingerprint closer to that of recovered subjects than to that of patients in the early stage of the infection; instead, the lipoproteome remains closer to that characteristic of the early stage infection for longer times.

## Results

### Cohort description

This study is based on a population of subjects hospitalized during the two first waves of the COVID-19 pandemic in Florence (i.e. before a significant spread of the Delta variant). The composition of this population reflects the incidence of the disease in terms of age and comorbidities that represent main risk factors (see **Tables 1** and S1 and S2) and does not include any vaccinated patient.

**Table 1:** EDTA-plasma samples included in the analysis.

Group	N° subjects	Age (mean ± SD)	Sex (F, M)
COVID-19≤21	246	67.45 ± 15.78	F = 108; M = 138
COVID-19>21	28	71.49 ± 15.41	F = 17; M = 11
Post COVID-19	95	65.56 ± 14.48	F = 42; M = 53

**Fig 1:**
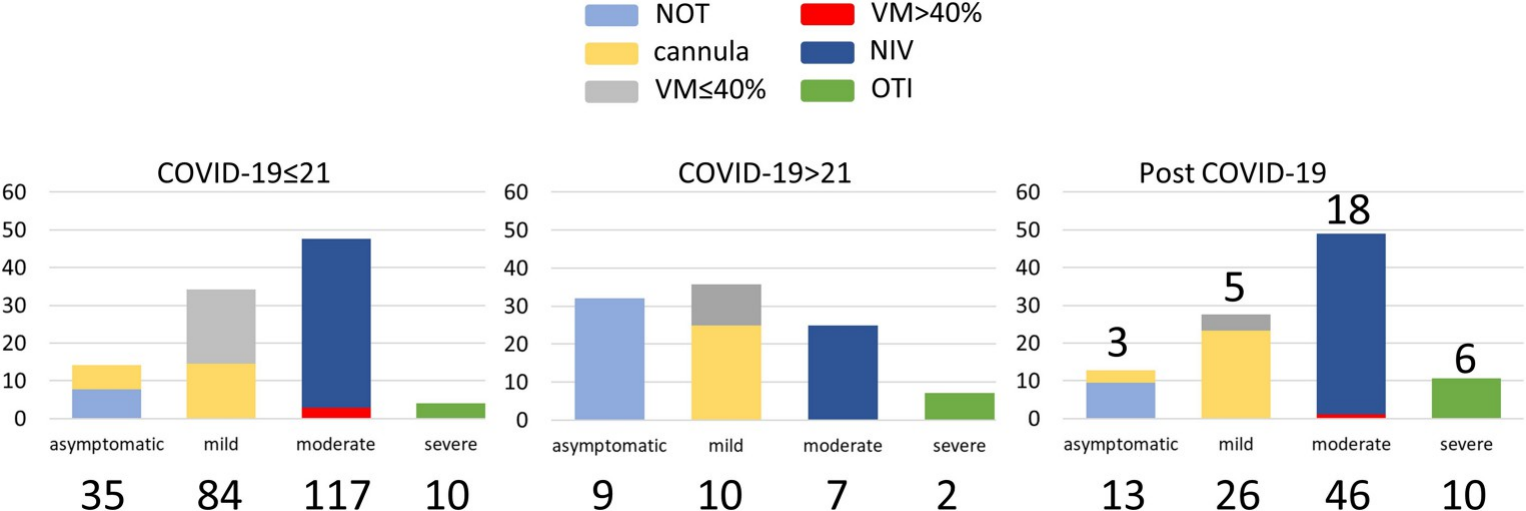
Clinical classification. Distribution (%) of the subjects according to the 4 descriptors defining disease severity in the acute phase, i.e. asymptomatic, mild, moderate and severe. Treatments are indicated by color code: no oxygen therapy (NOT; cyan), nasal cannula (yellow), Ventimask (VM, FiO2≤40%, grey), VM (FiO_2_>40%, red), non invasive ventilation (NIV, blue), orotracheal intubation (OTI, green). In the right panel, the numbers above each bar indicate the number of subjects with CT abnormalities at the follow-up visit. In all panels the absolute numbers of subjects are indicated below each bar.

A total of 369 human EDTA-plasma samples were used for the NMR analysis. Two hundred and seventy-four samples were collected from COVID-19 hospitalized patients. Among them, 246 samples were collected from COVID-19 positive patients within 21 days (mean 6.9 days) from clinical diagnosis (positive molecular nasopharyngeal swab for SARS-CoV-2 infection), hereafter named COVID-19≤21; 28 samples were collected from COVID-19 patients still positive beyond 21 days (mean 60.4 days, min 22 and max 139 days) from the clinical diagnosis (COVID-19>21). Ninety-five samples were obtained from recovered subjects (Post COVID-19), who had double negative swab since 2–6 months (mean 107.6 days, min 52 and max 182 days). For the last group samples were collected during follow-up monitoring. All samples were obtained from different individuals; the detailed demographic and clinical characteristics of the subjects are reported in **S1 and S2 Tables**. The subjects have been classified according to the disease severity in the acute phase, defined by the four descriptors reported in Materials and Methods. The COVID-19≤21 group is composed of 35 asymptomatic, 84 mild, 117 moderate and 10 severe subjects. The COVID-19>21 group includes 9 asymptomatic, 10 mild, 7 moderate, and 2 severe subjects. The COVID-19>21 at the moment of blood collection were asymptomatic or had only very mild symptoms. For the subjects included in the Post COVID-19 group, the classification at the moment of the acute infection was: 13 asymptomatic, 26 mild, 46 moderate and 10 severe subjects. All these figures are summarized in **Fig 1**. Most patients included in the study were hospitalized for COVID-19, even in the case of mild symptoms (according to the Italian guidelines during the initial phase of the pandemic) and the patients that were initially hospitalized for other reasons were a small minority with respect to the total subjects included in each of the three groups (S1 Table). At the moment of sample collection, for 32 out of 95 Post COVID-19 abnormalities were detect with computer tomography (CT), although not associated with pulmonary function problems. It is important to mention that we do not know if CT abnormalities were already present before SARS-CoV-2 infection or are a consequence of the disease.

### Early stage acute infection

#### Discrimination of COVID-19 vs. Post COVID-19 subjects based on the NMR fingerprint

**Fig 2:**
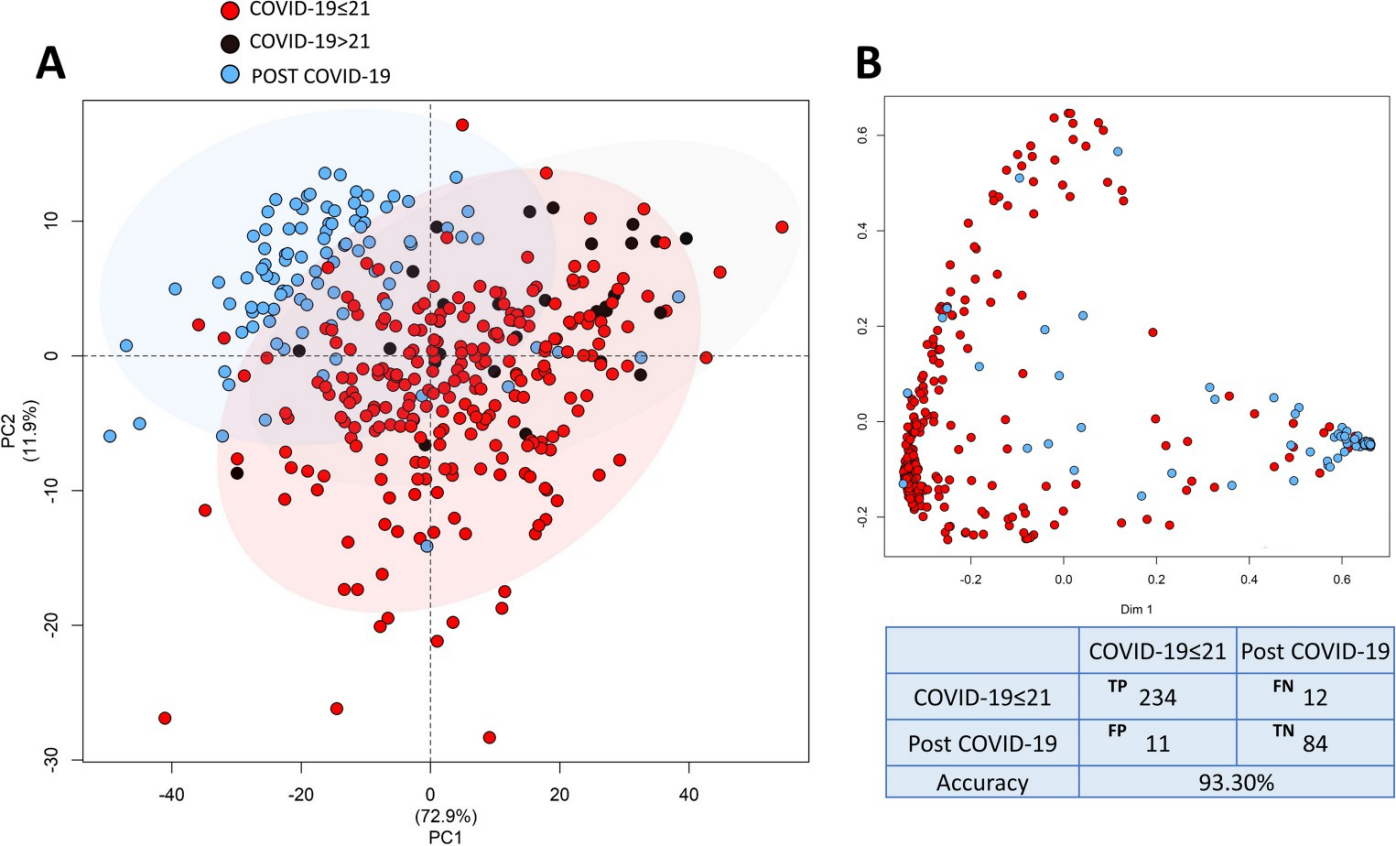
Multivariate statistics. A) Score plot of PCA analysis. Ellipsis indicate the 95% confidence intervals. B) Proximity plot of the RF model discriminating COVID-19≤21 patients and Post COVID-19 subjects with confusion matrix and accuracy value. In the confusion matrix, TP means true positive, FP false positive, TN true negative and FN false negative. Red dots: COVID-19≤21; black dots: COVID-19>21; blue dots: Post COVID-19.

Multivariate statistics was used to visualize and compare the EDTA-plasma metabolomic fingerprint of COVID-19 patients with respect to that of Post COVID-19 subjects, using the binned NMR spectra. The best results were obtained using NOESY spectra. Unsupervised Principal Component Analysis (PCA) was used to obtain a preliminary check of the data. The resulting score plot (**Fig 2A**) evidenced that a strong fingerprint of the disease exists, being COVID-19 samples and Post COVID-19 samples only partially overlapped. Random Forest (RF) was used as supervised method to compare and classify patients according to the diagnosis in the two extreme groups: COVID-19≤21 days and Post COVID-19 subjects. The RF model reported in **Fig 2B** shows that the NMR fingerprints of the two groups are significantly different and can be clearly discriminated from one another, with a RF discrimination accuracy of 93.3%.

Conversely, considering only the COVID-19≤21 days group, there are no clear-cuts among the four classes of disease severity in terms of metabolomic fingerprinting, with a discrimination accuracy between the two extreme classes (asymptomatic vs. severe) of 55.6%.

Analogously, the multivariate analysis did not revealed differences within the Post COVID-19 group between those who had CT abnormalities at the follow-up and those who did not (RF discrimination accuracy 55.8%).

#### Metabolite analysis in COVID-19 vs. Post COVID-19 subjects

**Fig 3:**
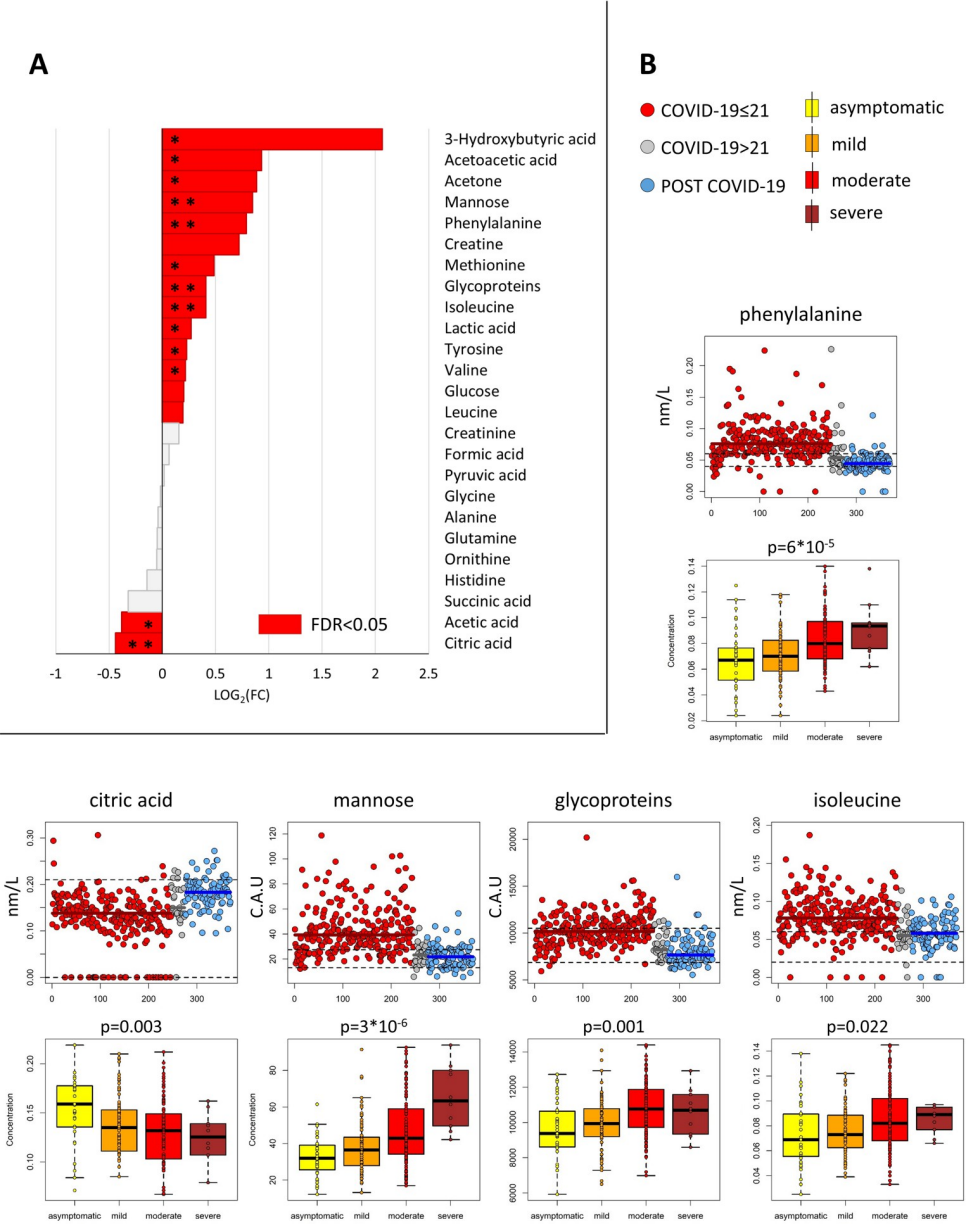
Metabolite profiling. A) Values of Log_2_Fold Change (FC) of quantified metabolites. Positive/negative values, have higher/lower concentration in serum samples from COVID-19≤21 patients with respect to Post COVID-19 subjects. Red bars refer to significantly different metabolites (p-value (FDR) <0.05); Cliff’s delta effect size are also reported: ** large, *medium. B) Upper panels: Scatter plots of concentration levels for significant metabolites (p-value (FDR) ≤0.05) with a “large” Cliff’s delta effect-size for the comparison COVID-19≤21 vs. Post COVID-19 groups; red dots represent COVID-19≤21 subjects, grey dots refer to COVID-19>21 subjects and blue dots to Post COVID-19 individuals; the median of each group is represented as a colored line; black dashed lines embrace the concentration ranges in a “healthy” population. Lower panels: boxplot of the concentration levels of COVID-19≤21 samples according to the grade of severity, i.e. asymptomatic (yellow), mild (orange), moderate (red), severe (brown).

Twenty-five metabolites were unambiguously identified in all the spectra. Their concentrations were analysed by univariate statistics to evaluate significant differences between COVID-19≤ 21 and Post COVID-19 groups (**Fig 3A**). Of these, 16 metabolites display significantly different levels between the two groups (**Fig 3A**). Particularly interesting are the high levels of phenylalanine, mannose, glycoproteins and isoleucine, along with a low level of citrate, in COVID-19 subjects. These metabolites are characterized by a large Cliff’s delta effect size. **Fig 3B** reports the concentrations for all these metabolites: in the COVID-19≤21 group, phenylalanine, isoleucine and mannose levels are outside the concentration ranges of a reference “healthy” population, while in the Post COVID-19 samples their concentrations fall inside the normal range. Moreover, the levels of these five metabolites in COVID-19≤21 samples significantly increase/decrease going from asymptomatic, to mild, moderate and severe subjects (**Fig 3B**). This trend is particularly evident for mannose.

The ketone bodies (3-hydroxybutyrric acid, acetoacetic acid and acetone) together with three amino acids (methionine, tyrosine, valine), lactic acid and acetic acid show significantly different concentrations between COVID-19≤21 and Post COVID-19 groups, and have a medium Cliff’s delta effect size, **Figs 3A** and S3.

No significant differences in metabolite concentrations are detected when comparing Post COVID-19 subjects with CT abnormalities vs. POST COVID-19 subjects with no CT abnormalities. Because of the small number of subjects with CT anomalies, these results should be confirmed on a larger cohort.

#### Lipoprotein analysis in COVID-19 vs. Post COVID-19 subjects

**Fig 4:**
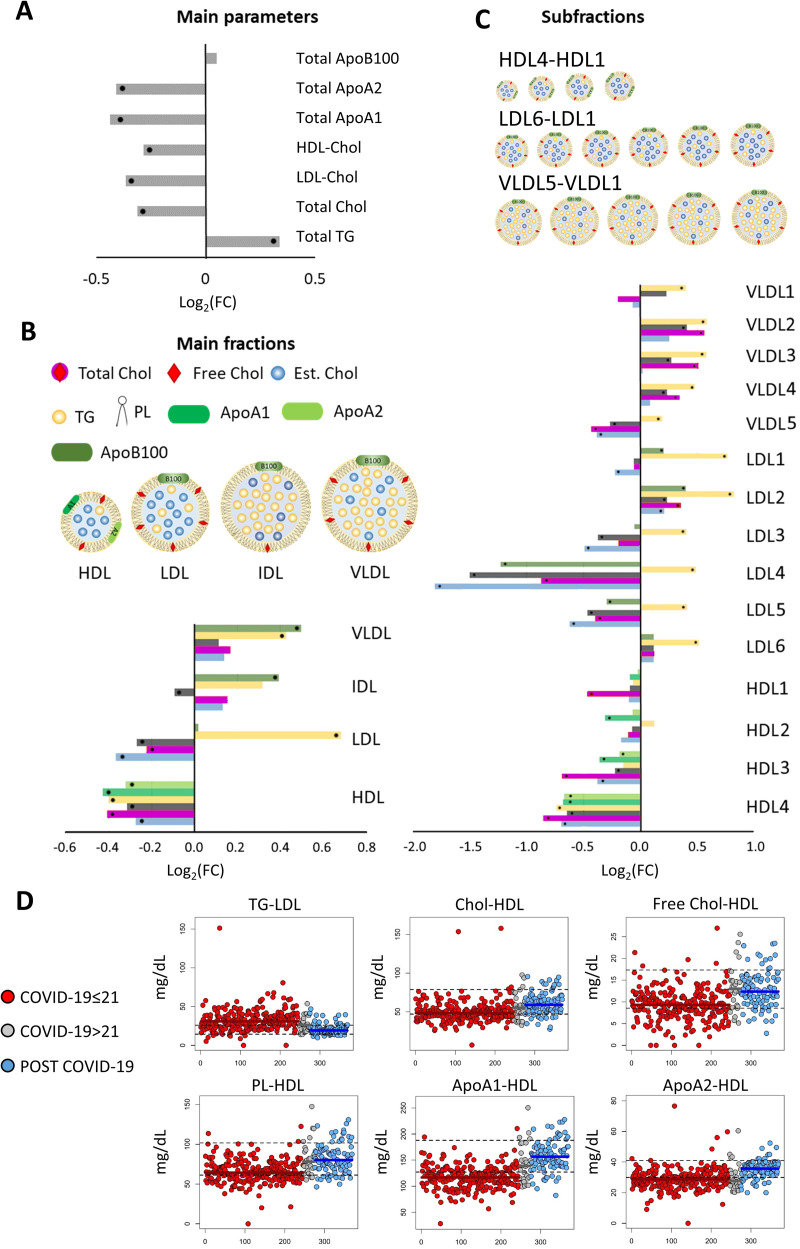
Lipoprotein profiling. Values of Log_2_ Fold Change (FC) of lipoprotein parameters. (A) Main parameters; (B) main fractions; (C) subfractions. Positive/negative values, have higher/lower concentration in serum samples from COVID-19≤21 patients with respect to Post COVID-19 subjects. Statistically significant parameters are marked with black dots. D) Scatter plots of concentration levels for significant main fraction parameters (p-value (FDR) ≤0.05) with a “large” Cliff’s delta effect-size for the comparison COVID-19≤21 vs. Post COVID-19 groups; red dots represent COVID-19≤21 subjects, grey dots refer to COVID-19>21 subjects and blue dots to Post COVID-19 individuals; the median of each group is represented as a colored line; black dashed lines embrace the concentration ranges in a “healthy” population.

One hundred twelve parameters, among lipoprotein main- and sub-fractions were analysed through the IVDr tool [25]. The COVID-19≤21 group has a strongly different lipoprotein profile with respect to that of Post COVID-19 group, with differences attributable to 74 parameters (**S3 Table** and **Fig 4**). In particular, the COVID-19 group is characterized by a general increment of triglycerides (TG), and a decrement of phospholipids (Ph), total cholesterol (Chol), HDL- and LDL-cholesterol, as well as of apolipoprotein A1 and A2 (ApoA1 and ApoA2). Looking in particular at the lipoprotein main- and sub- fraction composition, we detected a significant decrement of all the components associated to HDL (TG, Ph, Chol, Free Chol, ApoA1 and ApoA2), with HDL3 and HDL4 as the most affected subfractions; a decrement of Chol, free Chol and Ph were also monitored in all LDL subfractions but LDL2 and LDL6. All LDL subfractions, as well as all VLDL subfractions, are also characterized by a significant increment of the total content of TG, in particular for LDL1 and LDL2. **S3 Table** reports the FDR p-values and the effect-size measures for all quantified lipoprotein main- and sub-fraction parameters.

**Fig 4D** shows the scatter plots of the concentration levels of the lipoprotein main fraction composition parameters that, in the comparison between COVID-19≤21 and Post COVID-19 subjects, have a significant p-value and a large Cliff’s delta effect size (TG-LDL, Chol-HDL, Free Chol-HDL, PL-HDL, Apo A1-HDL and Apo A2-HDL), **S3 Table**. As in the case of metabolites, in the COVID-19≤21 group, the levels of these parameters are outside or on the boundary of the range of the reference “healthy” population, while in most of the Post COVID-19 samples they fall inside.

When analyzed in terms of the 4 classes of disease severity (**S4 Fig**), only the TG-LDL concentration changes going from asymptomatic, to mild, moderate and severe subjects (even if not significantly), with LDL1, LDL2 and LDL3 as the most affected (p<0.05) subclasses (**S5 Fig**). Three lipoprotein main fraction parameters, i.e. TG-HDL, Chol-LDL and ApoB-VLDL show significantly different concentrations between COVID-19≤21 and Post COVID-19 groups, and a medium Cliff’s delta effect size, **S6 Fig and S3 Table.** As for TG-LDL, a significant trend is observed when going from asymptomatic to severe only when considering the two subfractions Chol-LDL4 and Chol-LDL5 (**S7 Fig**). A similar behavior is observed for the FreeChol-LDL4 and FreeChol-LDL5 as well as for the PL-LDL4 and PL-LDL5 (**S7 Fig**). These observations indicate that changes associated with the disease severity exist even for those parameters that are associated to small Cliff’s effect size (and significant p-value) in the comparison with the Post COVID-19 group.

No significant differences in lipoprotein levels are detected within the Post COVID-19 group, independently of the presence of CT abnormalities.

### The COVID-19>21 patients

The RF model built on the comparison between COVID-19≤21 and Post COVID-19 has been used to predict the NMR spectra of COVID-19>21 subjects. Only 5 out of the 28 COVID-19>21 patients have been predicted as Post COVID-19; the 5 patients did not belong to any specific group of severity in acute phase nor had any specific symptoms at the moment of blood sampling. Interestingly, the distribution of metabolites’ levels of the COVID-19>21 group (grey dots in **Fig 3**) is closer to that of the Post-COVID-19 subjects. The opposite situation is encountered for the lipoproteins (grey dots in **Fig 4**). These observations lead to two main conclusions: i) the lipoproteome characteristic of the acute stage of the disease normalizes more slowly than the metabolome; ii) the ^1^H NMR spectral fingerprint of the acute infection is mainly determined by the lipoprotein components, and therefore the RF model predicts most of these patients as acute ones.

## Discussion

The pandemic of COVID-19 has been one of the worst health crises worldwide and there is a high interest in the identification of alterations of blood markers as indicators of pathophysiological changes caused by the viral infections. ^1^H NMR-based metabolomics has been used by several groups to profile the host responses to SARS-CoV-2 infection in humans by performing quantitative metabolomics and lipoproteomics of serum/plasma samples.

In our previous work, based on 30 hospitalized patients with severe COVID-19 symptoms (12 of them with invasive ventilation therapy) [6], we demonstrated that, despite the heterogeneity of the clinical symptoms, COVID-19 patients are characterized by a strong plasma metabolomic and lipidomic signatures that are significantly different from those of healthy subjects (91.7% and 87.5% accuracy, for metabolites and lipoproteins, respectively). In particular, 11 metabolites and 48 lipoprotein-related features displayed significant alterations between acute COVID-19 patients and sex and age matched healthy controls [6]. That study suffered by two main limitations: the small number of patients and the use of Ficoll as blood separator. The latter introduces broad resonances in the spectrum, thus reducing the number of quantifiable metabolites and lipoproteins. Here, using simple EDTA-plasma samples we were able to observe alterations of a larger number of metabolites and lipoproteins (16 and 74, respectively), largely matching previously identified markers of the disease. This finding is made robust by the use of a large cohort of patients (246 + 28) with different disease severity sampled at different time points after the first positive nasal swab in comparison with 95 Post COVID-19 subjects sampled 2–6 months after negative swab. As indicated by metabolites and lipoproteins in **S1 and S2 Figs**, our former and present findings are largely consistent with those obtained by other NMR research groups worldwide [6–14], demonstrating that they reflect authentic pathophysiological changes in response to COVID-19. This is not a trivial finding, as in studies of this type numerous confounding factors can arise from multiple clinical manifestations of the disease and different therapeutic treatments.

Coherent changes reported for the NMR spectra of the serum/plasma samples of COVID-19 patients are: i) higher levels of ketone bodies, of the amino acids phenylalanine, glutamate and valine, of the energetic molecules pyruvate, succinate, glucose, formate, creatine and mannose and of the glycoproteins (GlycA and GlycB); ii) lower levels of the amino acids glutamine, glycine and histidine; iii) higher level of the total TG content (both in VLDL, LDL and HDL fractions), of the ApoB100/ApoA1 ratio and of cholesterol, free cholesterol and phospholipids associated to VLDL along with the total number of VLDL particles; iv) lower levels of cholesterol, free cholesterol, PL and apolipoproteins (ApoA1 and ApoA2) associated to HDL and LDL fractions.

More ambiguous remains the behaviour of alanine, leucine and citrate, which show different trends in different studies, **S1 Fig**.

The metabolomic/lipoproteomic changes can be explained as the consequence of the complex interactions between the host and SARS-CoV-2, leading to the reprogramming of the host metabolism aimed at the energy supply for viral replication and for host immunological response. The dysregulation of the molecules pyruvate, succinate and glutamate, can be ascribed to an impairment of the central carbon metabolism in COVID-19 patients [6,7,26]. Accordingly, it has been reported that SARS-CoV-2 infection is characterized by a strong activation of gluconeogenesis to meet biosynthetic and bioenergetic demands [7,27]; this observation is in line with the observed decrement of the circulating gluconeogenic amino acids glycine, histidine and glutamine and with the elevation of glucose. Moreover, the high levels of triglycerides and triglycerides-rich lipoproteins (i.e VLDL) could be caused by the reduction of acetyl-CoA oxidation within the mitochondria; as a consequence, acetyl-CoA oxidation is redirected to the synthesis of ketone bodies, leading to the observed high levels of 3-hydroxybutyrric acid, acetoacetic acid and acetone [7,26,28]. Also the increment of mannose can be explained as a dysregulation of carbohydrate metabolism and mitochondrial function, and it has been proposed as a biomarker of the disease severity [27]. Phenylalanine concentration has been also proposed as a marker of the disease severity (as a consequence of increased inflammation) [29]. In line with these observations we found significantly higher levels of both mannose and phenylalanine going from mild to severe “COVID-19<21 days” patients. The elevation of serum glycoproteins reflects the strong inflammation of the patients; accordingly, GlycA and GlycB have been identified as NMR detectable robust biomarkers of inflammation [30,31].

Regarding the lipoproteome, several other (not metabolomic-based) studies have reported changes in lipid and lipoproteins profile associated with COVID-19 [32,33]. According to metabolomics, the most frequently reported changes are a decrement in total serum cholesterol (and of its main fractions, i.e. LDL and HDL cholesterol) and ApoA1 levels, along with the elevated triglycerides previously discussed. In this framework, the virus replication and assembly are processes that drastically change the host lipid metabolism and overuse cell lipid resources [32–36].

Here, we demonstrate in a large cohort of patients how NMR-based analyses of plasma can provide hints for the research community to better understand COVID-19-associated host responses, by identifying a pool of metabolites and lipoproteins parameters that collectively contribute to the definition of disease severity and, more importantly, allow us to follow the individual response along the healing path. Notably, while both metabolites and lipoprotein parameters distinguish the disease severity, the restoration of the “healthy” values does not occur at the same rate. In particular, the alterations in lipoproteins persist in positive subjects much longer than those in metabolites. The latter could therefore provide the timeliest sign of the individual response to clinical treatment or spontaneous healing from the infection.

## Materials and methods

### Ethics statement

The study was conducted in accordance with the Declaration of Helsinki. The study was approved by Comitato Etico Regionale per la Sperimentazione Clinica della Toscana—sezione Area Vasta Centro on 19/01/2021, code “18436_bio”. Written informed consent for inclusion was obtained from each subject before enrolment in the study.

### Patient recruitment and sample collection

Subjects were enrolled in the context of the COMETA project, funded by the Tuscany region, and were collected at two hospital premises of Azienda USL Toscana centro, in Florence (Italy). They comprise COVID-19 patients, with positive molecular nasopharyngeal swab for SARS-CoV-2 infection, at different stages of the disease progression and COVID-19 recovered subjects, for which the eradication of the virus was confirmed by double negative nasopharyngeal swab repeated at least 24 hours apart (**Tables 1 and** S1 **and** S2). For the latter group, blood was collected on the occasion of the follow-up visit, scheduled within 2–6 months from the first negative swab. None of the patients were vaccinated, and all of them were infected before the spread of the Delta variant.

The analyses were done on EDTA-plasma. All the samples were collected, processed and stored following the ISO standards (ISO 23118:2021), designed for high quality biological samples for metabolomic analysis [37,38]. During the course of the study, the samples were stored at −80°C in the repository of the da Vinci European Biobank, which offered a conservation service (daVEB, DOI:10.5334/ojb.af, https://www.unifi.it/vp-11370-da-vinci-european-biobank.html, Italy).

Four different descriptors were used to define the state of the patients in the acute phase of the infection, which was based on the severity of the respiratory symptoms, i.e.: i) asymptomatic: not requiring oxygen treatment, or not requiring supplemental oxygen with respect to the treatment in progress before infection; ii) mild: requiring oxygen treatment mask (Ventimask, VM) or nasal prongs (FiO_2_≤40%); iii) moderate: requiring non-invasive ventilation (NIV) or VM with high-flow oxygen (FiO_2_>40%); iv) severe: requiring orotracheal intubation (OTI). Blood withdrawal for COVID-19≤21 occurred before NIV/VM (FiO_2_>40%) or OTI.

### NMR analysis

NMR samples were prepared and recorded according to standard procedures [21,22]. Frozen EDTA-plasma samples were thawed at room temperature and shaken before use. A total of 350 μL of sodium phosphate buffer (70 mM Na_2_HPO_4_; 20% (v/v) ^2^H_2_O; 6.1 mM NaN_3_, 4.6 mM sodium trimethylsilyl [2,2,3,3−^2^H_4_] propionate (TMSP), pH 7.4) was added to 350 μL of each serum sample; the mixture was homogenized by vortexing for 30 s. A total of 600 μL of each mixture was transferred into a 5.00 mm NMR tube (Bruker BioSpin) for the analysis. ^1^H-NMR spectra for all samples were acquired at the Magnetic Resonance Center (CERM) of the University of Florence using a Bruker 600 MHz spectrometer (Bruker BioSpin) operating at 600.13 MHz proton Larmor frequency and equipped with a 5 mm PATXI ^1^H−^13^C−^15^N and ^2^H-decoupling probe including a z axis gradient coil, an automatic tuning-matching (ATM) and an automatic and refrigerated sample changer (SampleJet, Bruker BioSpin). A BTO 2000 thermocouple served for temperature stabilization at the level of approximately 0.1 K at the sample. Before measurement, samples were kept for 5 min inside the NMR probe head, for temperature equilibration at 310 K.

For each serum sample, three one-dimensional (1D) ^1^H NMR spectra were acquired with water peak suppression and different pulse sequences that allowed the selective observation of different molecular components:

1. a standard NOESY 1Dpresat (noesygppr1d.comp; Bruker BioSpin) pulse sequence (using 32 scans, 98,304 data points, a spectral width of 18,028 Hz, an acquisition time of 2.7 s, a relaxation delay of 4 s and a mixing time of 0.01 s). This pulse sequence is designed to obtain a spectrum in which both signals of metabolites and high molecular weight molecules (lipids and lipoproteins) are visible;
2. a standard CPMG (cpmgpr1d.comp; Bruker BioSpin) pulse sequence (using 32 scans, 73,728 data points, a spectral width of 12,019 Hz and a relaxation delay of 4 s). This pulse sequence is designed for the selective observation of small molecule components in solutions containing macromolecules;
3. a standard diffusion-edited (ledbgppr2s1d.comp; Bruker BioSpin) pulse sequence (using 32 scans, 98,304 data points, a spectral width of 18,028 Hz and a relaxation delay of 4 s), for the selective observation of macromolecule components in solutions containing small molecules.

### Spectral processing

Free induction decays were multiplied by an exponential function equivalent to a 0.3 Hz line-broadening factor before applying Fourier transform. Transformed spectra were automatically corrected for phase and baseline distortions and calibrated (glucose doublet at δ 5.24 ppm) using TopSpin 3.5 (Bruker BioSpin). Each spectrum in the region 10.00–0.2 ppm was segmented into 0.02 ppm chemical shift bins, with exclusion of both EDTA resonances (regions: 2.53–2.60, 2.68–2.73, 3.07–3.24, 3.58–3.64 ppm) and water signal (region: 4.40–5.00 ppm). The corresponding spectral areas were integrated using the AssureNMR software (Bruker, BioSpin).

### Statistical analysis

All the multivariate analyses were applied on binned spectra through the R software (R 3.0.2) using in-house scripts. Unsupervised Principal component analysis (PCA) was used as exploratory analysis to obtain a preliminary outlook of the data (visualization in a reduced space, presence of clusters or outliers). The Random Forest (RF) algorithm [39] was used for classification in the comparison between the different groups of samples; the R package ‘Random Forest’ was used to grow a forest of 1000 trees, using the default settings.; stratified samplings have been performed to adjust for the different group sizes and to ensure equal representation of unbalanced groups. Accuracy, sensitivity, and specificity of all calculated models were assessed according to the standard definitions.

Twenty-five metabolites, whose peaks in the CPMG spectra were well defined and resolved, were assigned and their concentrations analysed. The assignment procedure was performed using an ^1^H NMR spectra library of pure organic compounds (BBIOREFCODE, Bruker BioSpin), public databases, *e*.*g*. the Human Metabolome Database [40], storing reference ^1^H NMR spectra of metabolites, and using literature data when available. Metabolites were analysed using In Vitro Diagnostics research (IVDr) B.I.-Quant PS tool (Bruker, BioSpin). For metabolites not present in IVDr list, the respective areas were integrated using a R script in-house developed.

One hundred twelve parameters associated to lipoprotein main- and sub-fractions were also analysed through IVDr Lipoprotein Subclass Analysis B.I.-LISA tool (Bruker, BioSpin) [25].

The deviation range (mean ± SD) for each metabolite and lipoprotein has been calculated starting from a reference population of EDTA-plasma samples from 177 (86 males and 91 females) non COVID-19 (“healthy”) subjects with a mean age of 61.20 years ± 14.4 (SD); these data were already available at CERM.

For univariate analyses, the non-parametric Wilcoxon-Mann-Whitney test was used for the determination of the meaningful metabolites between COVID-19≤ 21 group and Post COVID-19 group. The Kruskal-Wallis test was used for the determination of meaningful metabolites among the COVID-19 ≤ 21 subjects with different grades of disease severity.

The p-values were adjusted for multiple testing using the false discovery rate (FDR) correction using the Benjamini-Hochberg method [41]; an adjusted p-value < 0.05 was considered statistically significant. The effect size (Ef) was also calculated [42] to aid in the identification of the meaningful signals giving an estimation of the magnitude of the separation between the different groups. The magnitude is assessed using the thresholds provided in Romano et al. [43], i.e. |Ef| < 0.147 “negligible”, |Ef| < 0.33 “small”, |Ef| < 0.474 “medium”, otherwise “large”. Pearson correlations, adjusted for FDR using BH methods, were also calculated.

## Supporting information

S1 Table Demographic and clinical characteristics of the subjects. **CAD:** Coronary Artery Disease; CHF: Congestive Heart Failure; DCM: Dilated Cardiomyopathy; T2DM: Type 2 Diabetes; CRI: Chronic Renal Insufficiency. * with respect to SARS-CoV-2 infection.
(XLSX)

S2 Table Statistics of the demographic and clinical characteristics of the subjects. CAD: Coronary Artery Disease; CHF: Congestive Heart Failure; DCM: Dilated Cardiomyopathy; T2DM: Type 2 Diabetes; CRI: Chronic Renal Insufficiency.
(XLSX)

S3 Table List of the lipoprotein main- and sub-fraction parameters quantified in plasma sample of COVID-19≤21 days and Post COVID-19 subjects. The p-values (FDR) and Cliff’s delta effect size are reported.
(XLSX)

S1 Fig Literature review for metabolites. Metabolites that are found significantly up- or down- regulated in COVID-19 patients with respect to healthy controls in refs 6–14 are indicated by red and blue cells, respectively. White cells correspond to metabolites that were not reported as significant or were not measured. In the last column, the results obtained in COMETA for the comparison of COVID-19≤21 e Post COVID-19 are provided for comparison purposes.
(TIF)

S2 Fig Literature review for lipoproteins. Lipoprotein parameters that are significantly found up- or down- regulated in COVID-19 patients with respect to healthy controls in refs 6–14 are indicated by red and blue cells, respectively. White cells correspond to metabolites that were not reported as significant or were not measured. In the last column, the results obtained in COMETA for the comparison of COVID-19≤21 e Post COVID-19 are provided for comparison purposes.
(TIF)

S3 Fig Metabolites with a medium Cliff’s Delta effect size. Upper panels: scatter plots of concentration levels for significant metabolites (p-value (FDR) ≤0.05) with a “medium” Cliff’s Delta effect-size for the comparison COVID-19≤21 vs. Post COVID-19 groups; red dots represent COVID-19≤21 subjects, grey dots refer to COVID-19>21 subjects and blue dots to Post COVID-19 individuals; the median of each group is represented as a colored line; black dashed lines embrace the reference range in a “healthy” population. Lower panels: boxplot of the concentration levels of COVID-19≤21 samples according to the grade of severity, i.e. asymptomatic (yellow), mild (orange), moderate (red), severe (brown).
(TIF)

S4 Fig Lipoprotein main fraction parameters with a large Cliff’s Delta effect size. Upper panels: scatter plots of concentration levels for significant lipoprotein main fraction parameters (p-value (FDR) ≤0.05) with a “large” Cliff’s Delta effect-size for the comparison COVID-19≤21 vs. Post COVID-19 groups; red dots represent COVID-19≤21 subjects, grey dots refer to COVID-19>21 subjects and blue dots to Post COVID-19 individuals; the median of each group is represented as a colored line; black dashed lines embrace the reference range in a “healthy” population. Lower panels: boxplot of the concentration levels of COVID-19≤21 samples according to the grade of severity, i.e. asymptomatic (yellow), mild (orange), moderate (red), severe (brown).
(TIF)

S5 Fig TG-LDL subfractions. Boxplot of the concentration levels of COVID-19≤21 samples according to the grade of severity, i.e. asymptomatic (yellow), mild (orange), moderate (red), severe (brown).
(TIF)

S6 Fig Lipoprotein main fraction parameters with a medium Cliff’s Delta effect size. Upper panels: scatter plots of concentration levels for significant lipoprotein main fraction parameters (p-value (FDR) ≤0.05) with a “medium” Cliff’s Delta effect-size for the comparison COVID-19≤21 vs. Post COVID-19 groups; red dots represent COVID-19≤21 subjects, grey dots refer to COVID-19>21 subjects and blue dots to Post COVID-19 individuals; the median of each group is represented as a colored line; black dashed lines embrace the reference range in a “healthy” population. Lower panels: boxplot of the concentration levels of COVID-19≤21 samples according to the grade of severity, i.e. asymptomatic (yellow), mild (orange), moderate (red), severe (brown).
(TIF)

S7 Fig LDL4 and LDL5 subfractions. Boxplot of the concentration levels of COVID-19≤21 samples according to the grade of severity, i.e. asymptomatic (yellow), mild (orange), moderate (red), severe (brown).
(TIF)
