# Supplementary figures and images for: Profiling metabolites and lipoproteins in COMETA, an Italian cohort of COVID-19 patients
Source: PLoS Pathog. 2022 Apr 21;18(4):e1010443. doi: 10.1371/journal.ppat.1010443 (PMC9022834; doi:10.1371/journal.ppat.1010443)

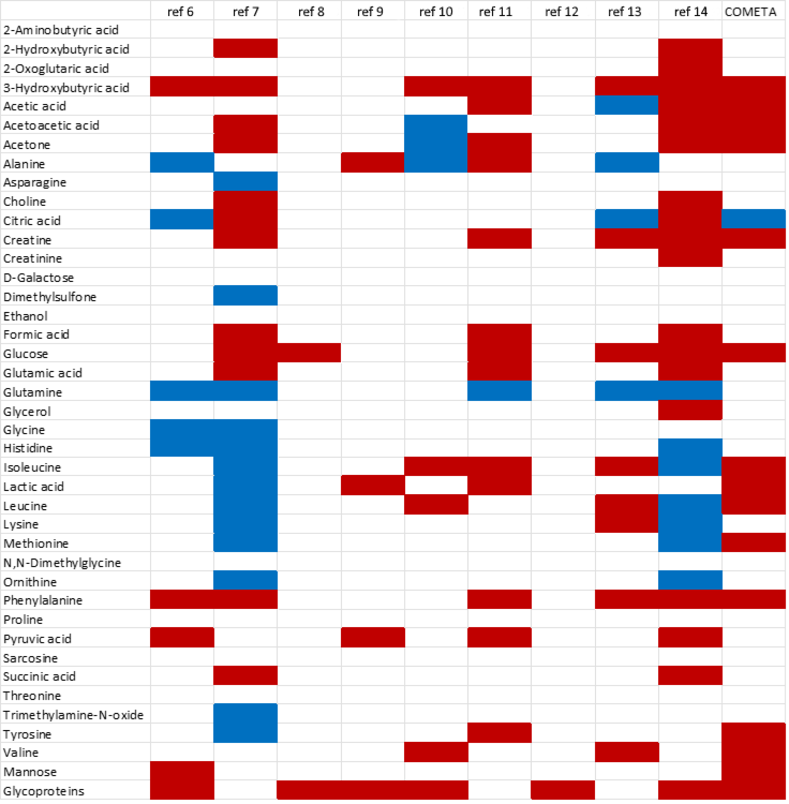

Supplement: S1 Fig — Metabolites that are found significantly up- or down- regulated in COVID-19 patients with respect to healthy controls in refs 6–14 are indicated by red and blue cells, respectively. White cells correspond to metabolites that were not reported as significant or were not measured. In the last column, the results obtained in COMETA for the comparison of COVID-19≤21 e Post COVID-19 are provided for comparison purposes. (TIF) [file ppat.1010443.s004.tif]

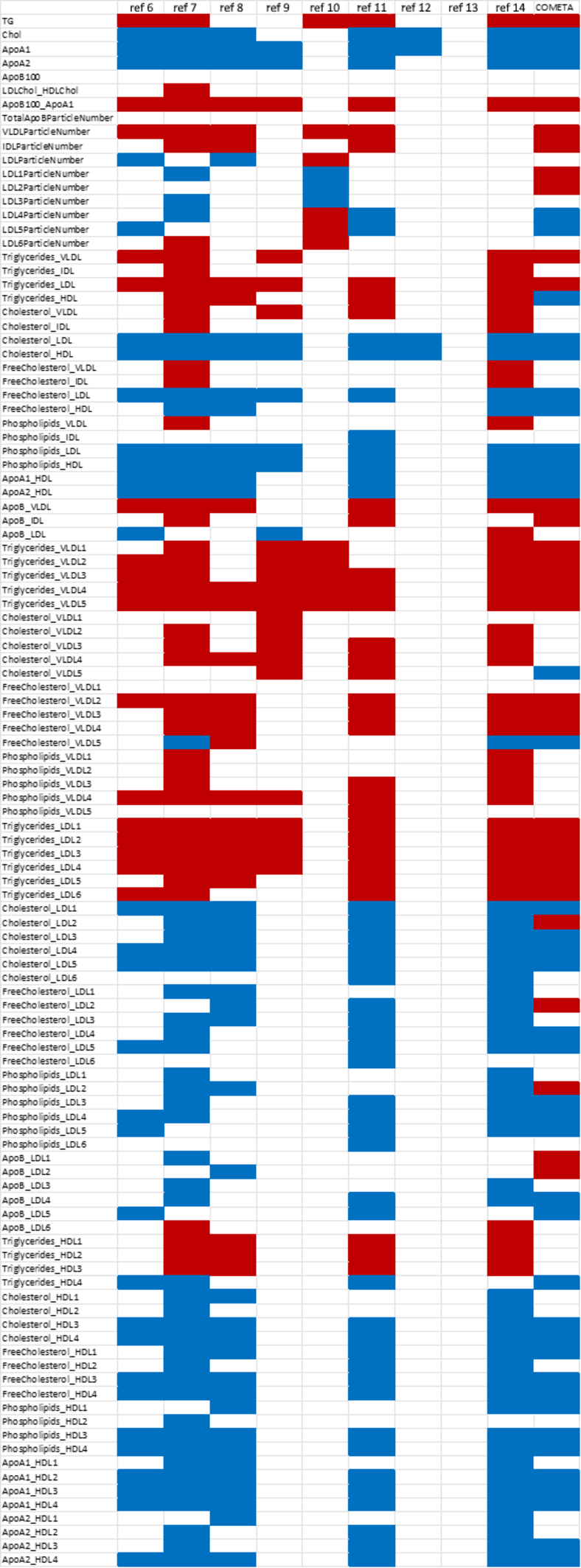

Supplement: S2 Fig — Lipoprotein parameters that are significantly found up- or down- regulated in COVID-19 patients with respect to healthy controls in refs 6–14 are indicated by red and blue cells, respectively. White cells correspond to metabolites that were not reported as significant or were not measured. In the last column, the results obtained in COMETA for the comparison of COVID-19≤21 e Post COVID-19 are provided for comparison purposes. (TIF) [file ppat.1010443.s005.tif]

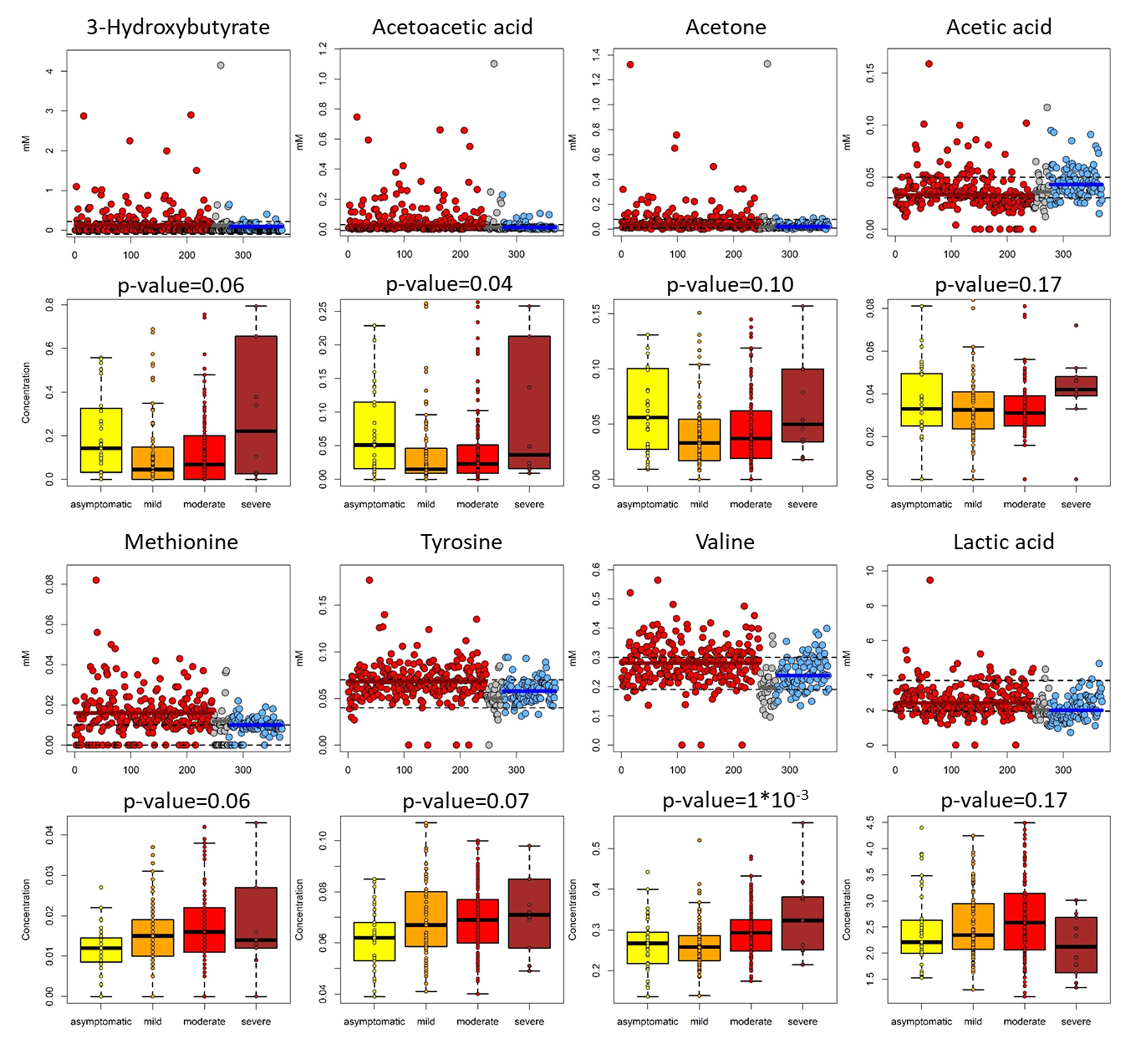

Supplement: S3 Fig — Upper panels: scatter plots of concentration levels for significant metabolites (p-value (FDR) ≤0.05) with a “medium” Cliff’s Delta effect-size for the comparison COVID-19≤21 vs. Post COVID-19 groups; red dots represent COVID-19≤21 subjects, grey dots refer to COVID-19>21 subjects and blue dots to Post COVID-19 individuals; the median of each group is represented as a colored line; black dashed lines embrace the reference range in a “healthy” population. Lower panels: boxplot of the concentration levels of COVID-19≤21 samples according to the grade of severity, i.e. asymptomatic (yellow), mild (orange), moderate (red), severe (brown). (TIF) [file ppat.1010443.s006.tif]

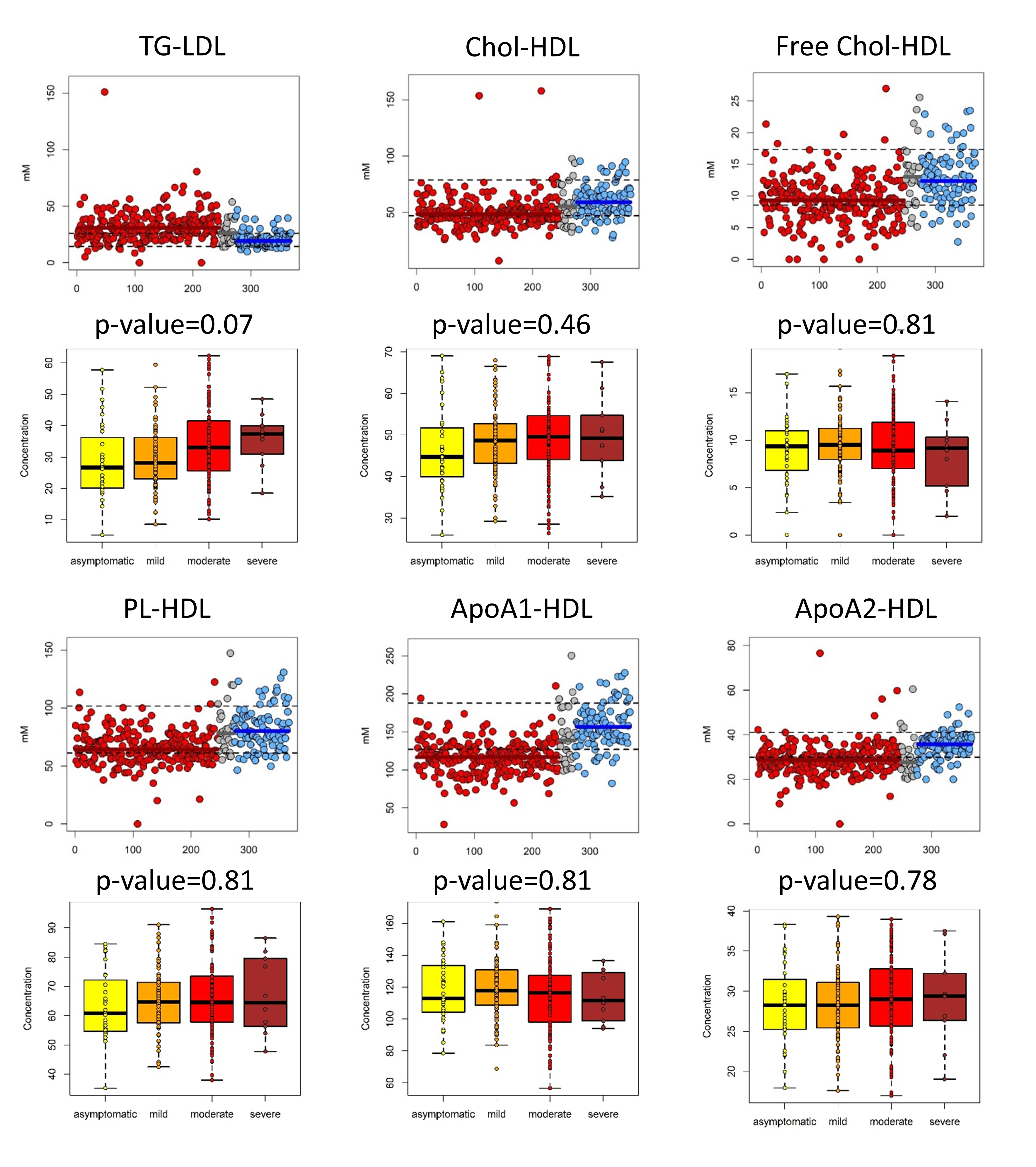

Supplement: S4 Fig — Upper panels: scatter plots of concentration levels for significant lipoprotein main fraction parameters (p-value (FDR) ≤0.05) with a “large” Cliff’s Delta effect-size for the comparison COVID-19≤21 vs. Post COVID-19 groups; red dots represent COVID-19≤21 subjects, grey dots refer to COVID-19>21 subjects and blue dots to Post COVID-19 individuals; the median of each group is represented as a colored line; black dashed lines embrace the reference range in a “healthy” population. Lower panels: boxplot of the concentration levels of COVID-19≤21 samples according to the grade of severity, i.e. asymptomatic (yellow), mild (orange), moderate (red), severe (brown). (TIF) [file ppat.1010443.s007.tif]

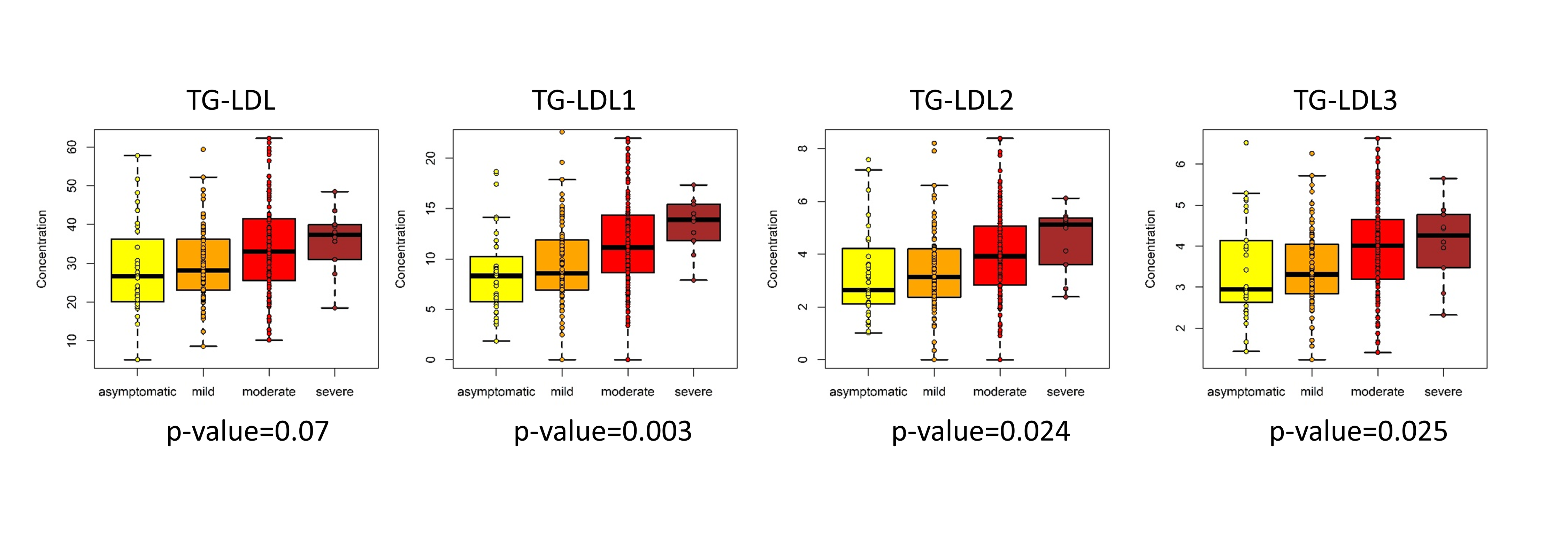

Supplement: S5 Fig — Boxplot of the concentration levels of COVID-19≤21 samples according to the grade of severity, i.e. asymptomatic (yellow), mild (orange), moderate (red), severe (brown). (TIF) [file ppat.1010443.s008.tif]

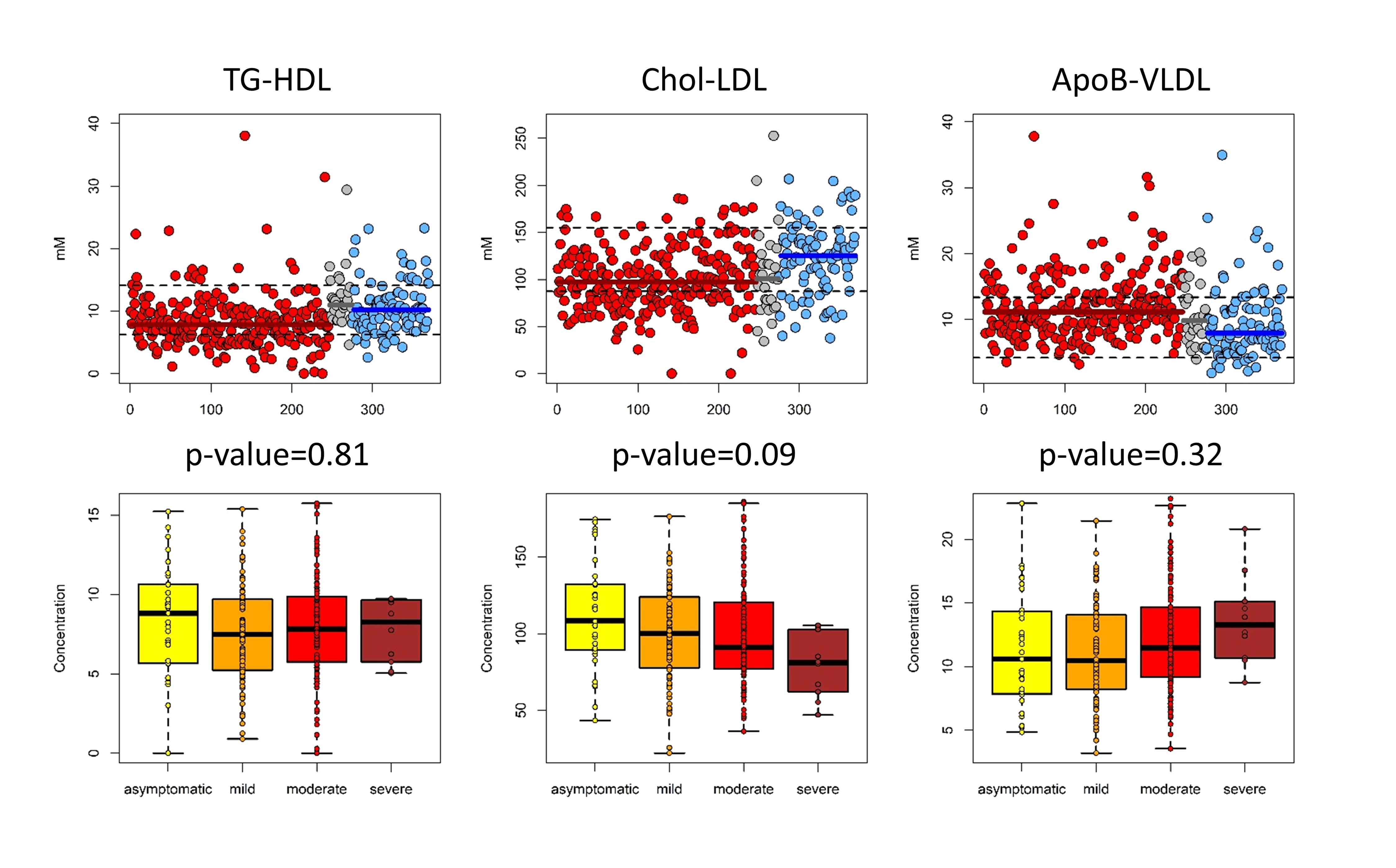

Supplement: S6 Fig — Upper panels: scatter plots of concentration levels for significant lipoprotein main fraction parameters (p-value (FDR) ≤0.05) with a “medium” Cliff’s Delta effect-size for the comparison COVID-19≤21 vs. Post COVID-19 groups; red dots represent COVID-19≤21 subjects, grey dots refer to COVID-19>21 subjects and blue dots to Post COVID-19 individuals; the median of each group is represented as a colored line; black dashed lines embrace the reference range in a “healthy” population. Lower panels: boxplot of the concentration levels of COVID-19≤21 samples according to the grade of severity, i.e. asymptomatic (yellow), mild (orange), moderate (red), severe (brown). (TIF) [file ppat.1010443.s009.tif]

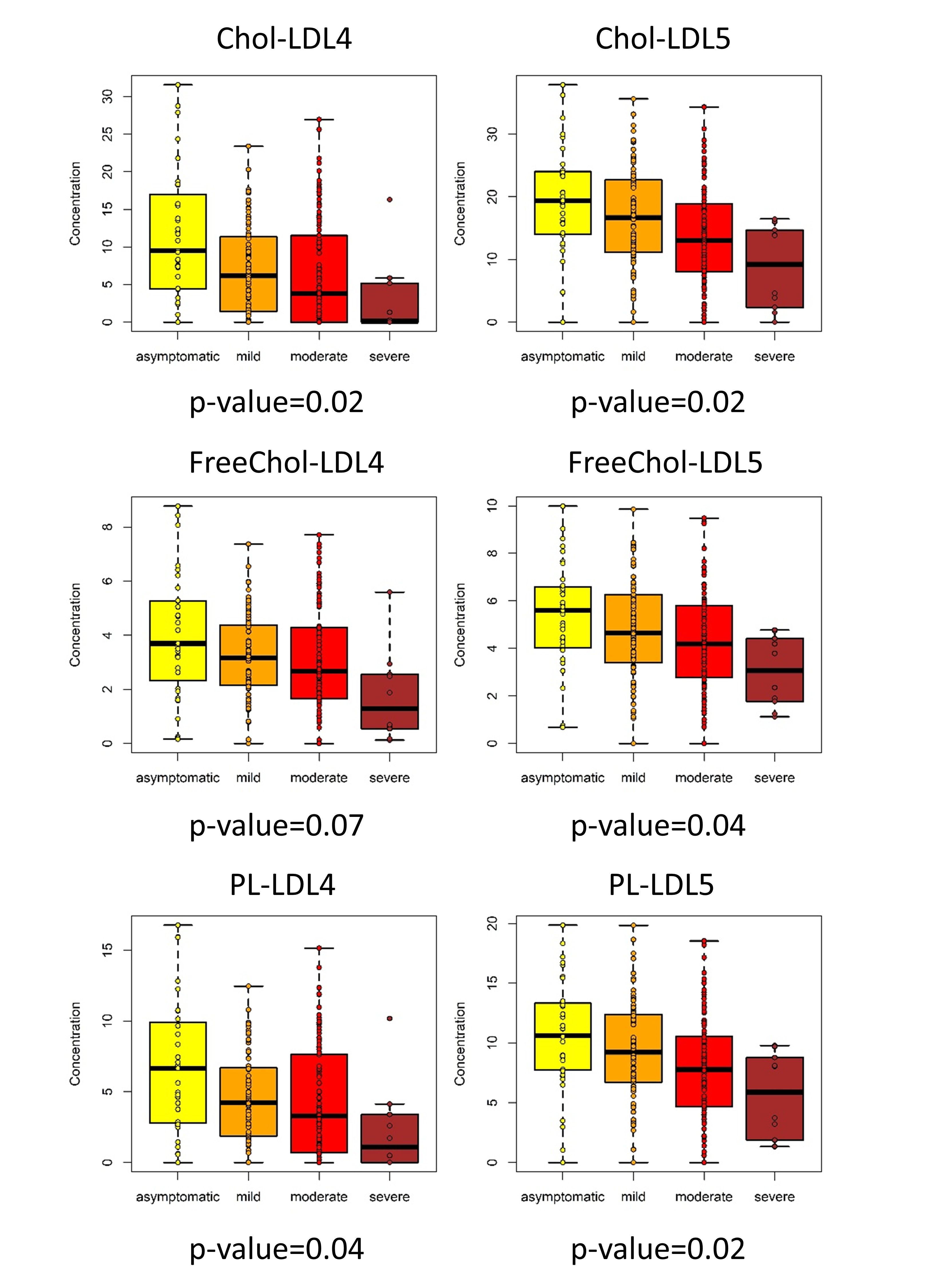

Supplement: S7 Fig — Boxplot of the concentration levels of COVID-19≤21 samples according to the grade of severity, i.e. asymptomatic (yellow), mild (orange), moderate (red), severe (brown). (TIF) [file ppat.1010443.s010.tif]
